# Associations of Self-Care Health Behaviors With Driving Cessation Among Older Drivers

**DOI:** 10.3389/fpubh.2022.794639

**Published:** 2022-03-24

**Authors:** Thelma J. Mielenz, Adam M. Whalen, Qian-Li Xue, Howard Andrews, Lisa J. Molnar, David W. Eby, Guohua Li

**Affiliations:** ^1^Department of Epidemiology, Mailman School of Public Health, Columbia University Irving Medical Center, New York, NY, United States; ^2^Department of Medicine, School of Medicine, Johns Hopkins University, Baltimore, MD, United States; ^3^Department of Biostatistics, Mailman School of Public Health, Columbia University Irving Medical Center, New York, NY, United States; ^4^Transportation Research Institute, University of Michigan, Ann Arbor, MI, United States; ^5^Department of Anesthesiology, Vagelos College of Physicians and Surgeons, Columbia University Irving Medical Center, New York, NY, United States

**Keywords:** health behavior, older adult, self-care, driving cessation, physical activity, sleep, community engagement

## Abstract

Older adults are at risk of driving cessation as they age, which can result in negative health outcomes including loss of independence. This study aimed to investigate the associations of self-care health behaviors with the risk of driving cessation. Demographics, health and driving characteristics were captured from healthcare systems in Denver, CO, San Diego, CA, Ann Arbor, MI, Baltimore, MD and Cooperstown, NY for 2,990 adults at baseline then followed from July 2015 to January 2021 *via* in-person assessments and questionnaires. The follow-up accumulated a total of 7,348 person-years and 46 driving cessations, yielding an incidence rate of 0.63 per 100 person-years. Multivariable Cox proportional hazards regression was used to evaluate the relationship between self-care behaviors and driving cessation, stratified by gender, and accounting for multiple failure events and clustering by study site. Ability to participate in social roles and activities was associated with an 8% reduction in the risk of driving cessation [adjusted hazard ratio (HR): 0.92; 95% CI: 0.89, 0.94]. Increased participation in social activities and relationships is associated with driving longevity in older adults and should be targeted for interventions to maintain driving mobility.

## Introduction

The majority of older adults starton a downward spiral toward driving cessation due to physical and cognitive declines (1). Self-care behaviors identified to slow these physical and cognitive declines, include: physical exercise, sleep hygiene, financial fitness, social interaction, and healthy relationships (2, 3). Low levels of physical activity and exercise are related to driving cessation (4). Sleep disturbances are associated with a decrease in annual mileage driven (5). Low levels of community engagement have been associated with driving cessation, yet the directionality of this relationship remains unclear (4). Self-care behaviors are modifiable by behavior change thus are important to investigate further for their effects on driving cessation. Using longitudinal data from a multisite prospective cohort of 2,990 older drivers, we assessed the associations of baseline self-care behaviors (physical activity, community engagement, and sleep) with driving cessation. We hypothesized that healthy self-care behaviors can contribute to driving longevity over time and that there is a differential risk decrease depending on the self-care behavior. More specifically, we anticipated that physical activity would have the strongest effect, followed by community engagement and sleep health.

## Methods

### Study Design

The study design and methods for the LongROAD study are described in detail elsewhere (6). LongROAD consists of five US clinical sites with each site enrolling roughly 600 participants, with a total enrollment of 2,990 active drivers aged 65–79 at baseline. Enrollment began on July 6, 2015 and it was completed March 31, 2017. This study involved an analysis of data from baseline through the second anniversary. Driving cessation data were current as of 1/20/2021.

### Measures

#### Self-Care Behaviors

Three self-care behaviors were included: (1) social roles and activities to capture community engagement, (2) physical activity, and (3) sleep disturbance to capture sleep hygiene.

#### Social Health

We used the PROMIS® v2.0—Ability to Participate in Social Roles and Activities Short-Form (SF) 4a (a higher *T*-score reflects better and a lower *T*-score reflects worse) as the measure of community engagement.

#### Sleep Disturbance

We measured sleep disturbance using the PROMIS® v1.0—Sleep Disturbance SF 4a measure which, when compared with two legacy measures, Epworth Sleepiness Scale and the Pittsburgh Sleep Quality Index, was found to be precise and efficient (7).

#### Physical Activity

Our assessment of physical activity was adapted from the Modified Minnesota Leisure Time Activities Questionnaire (MLTA) (8). If subjects were in the lowest quintile by gender for kilocalorie expenditure per week as reported by six questions on the Minnesota Leisure Time Activities (MLTA) Questionnaire, then they were considered low physical activity (8).

#### Driving Cessation

Information regarding driving cessation were collected during follow-up telephone interviews 1–3 months after GPS data transmission from the DataLogger has ceased. The Driving Habits Questionnaire (DHQ), Oregon Older Driver survey, Candrive, and Advanced Driving Decisions and Patterns of Travel (ADDAPT) Questionnaire were adapted for our driving cessation survey (9). If a specific date of cessation was not available, date of cessation was considered the date on which the interview was conducted.

#### Demographics and Covariates

Besides considering demographics, two important potentially modifiable factors that are known to be associated with driving cessation, vision and cognition, were included. Self-reported vision was categorized as poor to good, very good, and excellent. Episodic and working memory was determined by Immediate and Delayed Word Recall (10). Correct word recall scores of 0–10 were considered impaired cognitive performance, and scores of 11–20 were considered unimpaired.

### Statistical Analysis

The primary goal of this analysis was a time-to-event analysis of the three self-care health behaviors on driving cessation. We assessed the proportional hazards (PH) assumption and if it was violated, then we conducted a stratified Cox model. We used the Efron method for tied events. Because it is possible for subjects to cease driving, then resume driving, and cease again, we first checked the failure data to see if this occurred. For this study, the self-care behaviors and covariate measures were assessed only at baseline as time-invariant predictors.

All statistical investigations and analyses were conducted using Stata SE version 16.1 (StataCorp. 2019. College Station, Texas: StataCorp LLC).

## Results

The mean scores for self-reported participation in social roles and sleep disturbance were 57.5, and 45.5, respectively, and 29% of respondents were considered low physical activity. As of January 20, 2021, the most recent date for which data were available, 46 subjects had ceased driving. Examination of cessation data showed that all subjects ceased and did not resume driving except for one, who ceased driving temporarily then resumed, but did not have a subsequent cessation event. The analysis accounted for the potential for multiple failures and subsequent resumption of at-risk person-time. The follow-up (*N* = 2,774) accumulated a total of 7,348 person-years, yielding an incidence rate of 0.63 per 100 person-years.

We conducted stratified Cox models to account for non-proportionality by gender. Education was dropped using the change-in-estimate method. The final stratified Cox model (*N* = 2,687) contained physical activity, participation in social activities, and sleep disturbance adjusted for our baseline demographics and covariates including age, marital status, cognition, and vision, accounting for error clustering by study site (Table 1).

**Table 1:** Adjustedhazard ratio of self-care behaviors with time to driving cessation with 95% confidence interval.

	Adjusted^*^
Physical activity	1.20 (0.67, 2.15)
Sleep disturbance	0.99 (0.95, 1.02)
Ability to participate in social roles and activities	0.92 (0.89, 0.94)

* *Adjusted for baseline age, marital status, vision, and cognitive health, stratified by gender, and clustered by site. Total N = 2,744 and 6,736.27 person-years*.

The adjusted hazard ratios (HRs) of driving cessation were 1.20 (95% CI: 0.67, 2.15) for physical activity, 0.99 (95% CI: 0.95, 1.02) for sleep disturbance, and 0.92 (95% CI: 0.89, 0.94) for the PROMIS® measure Ability to Participate in Social Roles and Activities (a higher score on the scale represents greater social participation ability). Therefore, every 1-unit increase (or 1/10th of a standard deviation on a *T*-score metric) in self-rated ability to participate in social roles and activities was associated with an 8% reduction in the risk of driving cessation.

## Discussion

Initially, it had been hypothesized that of the three self-care behaviors measured, physical activity would be the main effect driving the association (4). However, participation in social roles and activities suggested a modest protective effect on driving cessation after adjusting for other self-care health behaviors and covariates. In other words, greater community engagement can result in better health driving outcomes for aging drivers. This finding is particularly salient in light of the ongoing COVID-19 pandemic. Among older adults in the US, considered one of the most vulnerable groups to the new disease, loss of community engagement due to public health containment measures may be associated with a subsequent loss of independence through driving (11).

Others in the literature report on the role that social participation plays on driving cessation. Prior to driving and after cessation social engagement declines (12). Older drivers who are socially active and provide rides to activities to family and friends avoid driving cessation longer (13–15). A strength of this study is measuring social roles and activities with a precise PROMIS measure compared to Pachana et al. (16) who measured social engagement as a dichotomous variable (17).

The number of participants who stopped driving over the 3-year period may be low at 46 of the 2,687 included in the final analysis. Self-reported reasons for driving cessation were not taken into account. Self-care health behaviors and all other covariates were assessed only at baseline. It is possible that self-care behaviors may be time-variant. We limited the number of modifiable and other covariates we controlled for to focus on the self-care behaviors and non-modifiable demographics.

The chosen behaviors were selected because they are modifiable on the individual level, at least to a certain extent. If the evidence presented here is confirmed, interventions may include targeted outreach campaigns for older adults to keep them engaged in the community in meaningful ways.

## Public Health Implications

An increased ability to participate in social roles and activities was moderately associated with a reduction in cessation risk adjusting for other self-care health behaviors and covariates. Further research is needed in this area to better understand these associations, and how interventions can be applied to support aging drivers and reduce the negative effects of driving cessation.

## Data Availability Statement

The original contributionspresented in the study are included in the article/Supplementary Material, further inquiries can be directed to the corresponding author/s.

## Ethics Statement

The research was approved andmonitored by the Institutional Review Board of the Columbia University Medical Center [IRB-AAAN9950]. The patients/participants provided their written informed consent to participate in this study.

## Author Contributions

TM: conceptualization and supervision. TM, AW, Q-LX, HA, LM, DE, and GL: methodology. AW and TM: formal analysis and writing—original draft preparation. TM, HA, LM, DE, and GL: data curation. AW, DE, LM, GL, HA, Q-LX, and TM: writing—review and editing. All authors have read and agreed to the published version of the manuscript.

## Funding

This work was supported by the AAA Foundation for Traffic Safety. This research was supported in part by Grant 1 R49 CE002096-01 from the Centers for Disease Control and Prevention, National Centerfor Injury Prevention and Control to the Center for Injury Epidemiology and Prevention at Columbia University. Its contents are solely the responsibility of the authors and do not necessarily represent the official views of the Centers for Disease Control and Prevention.

## Author Disclaimer

The contents are solely the responsibility of the authors and do not necessarily represent the official views of the Centers for Disease Control and Prevention.

## Conflict of Interest

The authors declare that the research was conducted in the absence of any commercial or financial relationshipsthat could be construed as a potential conflict of interest.

## Publisher's Note

All claims expressed in this article are solely those of the authors and do not necessarily represent those of their affiliated organizations, or those of the publisher, the editors and the reviewers. Any product that may be evaluated in this article, or claim that may be made by its manufacturer, is not guaranteed or endorsed by the publisher.
