# Supplementary material for: Associations of Self-Care Health Behaviors With Driving Cessation Among Older Drivers
Source: Front Public Health. 2022 Mar 24;10:794639. doi: 10.3389/fpubh.2022.794639 (PMC8987349; doi:10.3389/fpubh.2022.794639)
Supplement: Supplementary file 1 [file Table_1.DOCX]

**Supplemental Tables**

| Table 2.  Baseline characteristics of the LongROAD study population  (N = 2,990) | |
| --- | --- |
|  | N (%) |
| Gender |  |
| Male | 1403 (46.92) |
| Female | 1587 (53.08) |
| Age |  |
| 65-69 | 1243 (41.57) |
| 70-74 | 1037 (34.68) |
| 75-79 | 710 (23.75) |
| Race/Ethnicity (N = 2,986) |  |
| White, NH^1^ | 2557 (85.63) |
| Black, NH | 212 (7.10) |
| Hispanic | 81 (2.71) |
| Asian | 66 (2.21) |
| American Indian | 18 (0.16) |
| Alaska Native, Native Hawaiian, Pacific Islander | 3 (0.10) |
| Other, NH | 49 (1.64) |
| Marital Status (N = 2,962) |  |
| Married/Living with Partner | 1975 (66.68) |
| Divorced/Separated/Never Married/Widowed | 987 (33.32) |
| Education Level (N = 2,981) |  |
| High School Diploma or Less | 336 (11.27) |
| Some College, Associate’s Degree, Vocational/Trade School | 726 (24.35) |
| Bachelor’s Degree | 698 (23.41) |
| Advanced Degree | 1221 (40.96) |
| Word Recall (N = 2,905) |  |
| Impaired Cognition (0-10 correct) | 1504 (51.77) |
| Unimpaired Cognition (11-20 correct) | 1401 (48.23) |
| Self-Rated Vision  (N = 2,988) |  |
| Excellent | 750 (25.10) |
| Very good | 1255 (42.00) |
| Poor to good | 983 (32.90) |
| Ability to Participate in Social Roles and Activities (N = 2,923) |  |
| Mean (SD) | 57.45 (6.87) |
| Sleep Disturbance (N = 2,979) |  |
| Mean (SD) | 45.49 (8.21) |
| Physical activity (N = 2,876) |  |
| Normal activity | 2051 (71.31) |
| Low activity | 825 (28.69) |

^1^NH: non-Hispanic; ^2^SD: standard deviation

**Table 3**: Assessment of proportionality of survival functions for self-care behaviors and covariates

|  | Log-Rank Test^1^ | | | Schoenfeld Residual Test^2^ | | | |
| --- | --- | --- | --- | --- | --- | --- | --- |
|  | $\chi^{2}$ | df | p-value | $\rho$ | $\chi^{2}$ | df | p-value |
| Gender^3^ | 0.11 | 1 | 0.7445 | -0.4936 | 10.05 | 1 | 0.0015 |
| Age | 13.87 | 2 | 0.001 | 0.2140 | 1.84 | 1 | 0.1745 |
| Marital status | 6.51 | 1 | 0.0107 | 0.0248 | 0.02 | 1 | 0.8791 |
| Education^4^ | 1.88 | 3 | 0.5986 | -0.3301 | 4.36 | 1 | 0.0368 |
| Word recall | 4.17 | 1 | 0.0411 | -0.0772 | 0.24 | 1 | 0.6264 |
| Vision | 7.28 | 2 | 0.0263 | -0.0333 | 0.05 | 1 | 0.8311 |
| Physical activity | 1.46 | 1 | 0.227 | -0.1344 | 0.69 | 1 | 0.4046 |
| Social roles | -- | -- | -- | 0.1527 | 0.74 | 1 | 0.3885 |
| Sleep disturbance | -- | -- | -- | 0.0918 | 0.35 | 1 | 0.5525 |
| Global test | -- | -- | -- | -- | 20.52 | 9 | 0.0150 |

^1^ Log-rank tests were only performed for categorical variables

^2^ Initial Schoenfeld residual assessment without any stratification or error clustering adjustment

^3^ In the final model, the data was stratified by gender to account for non-proportionality

^4^ Education was dropped in the final model due to violation of the proportional hazards assumption and change-in-estimate model iteration
